# Insensitivity to atorvastatin is associated with increased accumulation of intracellular lipid droplets and fatty acid metabolism in breast cancer cells

**DOI:** 10.1038/s41598-018-23726-3

**Published:** 2018-04-03

**Authors:** Barbara Lettiero, Maria Inasu, Siker Kimbung, Signe Borgquist

**Affiliations:** 1 Division of Oncology and Pathology, Department of Clinical Sciences, Lund University, Lund, Sweden; 2 Clinical Trial Unit, Skåne University Hospital, Lund, Sweden

## Abstract

Apart from the relevant lipid-lowering effects, statins have demonstrated significant, although heterogeneous, anti-tumor activities in preventing breast cancer (BC) progression. To characterize the critical pathways behind the diverse responses to therapy, we investigated statin-induced changes in regulation of lipid metabolism and abundance of neutral lipid-containing cytoplasmic lipid droplets (LDs) in BC cells displaying different sensitivity to atorvastatin. Following atorvastatin treatment, accumulated LD levels inversely mirrored the marginal anti-proliferative effects in a dose and time-dependent manner in the less-sensitive BC cells. Transcriptional profiling excluded dysregulation of lipid uptake and efflux as specific mechanisms associated with differences in LD accumulation and anti-proliferative effects of atorvastatin. Notably, significant upregulation of genes involved in unsaturated fatty acid metabolism [stearoyl-CoA desaturase (*SCD*)] and cholesterol biosynthesis [3-hydroxy-3-methylglutaryl-CoA reductase (*HMGCR*)], were associated with atorvastatin insensitivity. Taken together, the increased ability to store neutral lipids in LDs as consequence of atorvastatin treatment likely confers a proliferative advantage to BC cells and may serve as potential biomarker of statin resistance in BC. Contributions of cholesterol biosynthesis and unsaturated fatty acid metabolism to LD formation should be thoroughly explored for better understanding of the molecular mechanisms underlying statin-induced effects against BC progression.

## Introduction

Statins are potent inhibitors of 3 Hydroxy-3-Methylglutaryl-CoA Reductase (*HMGCR*), the critical enzyme in the mevalonate pathway, leading to cholesterol biosynthesis^1^. Nowadays statins are widely prescribed as therapeutic agents to treat hypercholesterolemia and prevent cardiovascular diseases^2^. In the last decades, the lipophilic class of statins has attracted considerable attention because of the conclusive epidemiological findings supporting a role in preventing breast cancer recurrence^3,4^. Accordingly, accumulated clinical data^5–7^ and preclinical studies^5,8–10^ revealed statin-induced anti-proliferative and pro-apoptotic effects on primary breast tumors and breast cancer (BC) cell lines. However, the responses to statin treatment observed in both preclinical models and breast cancer patients are heterogeneous^5,6,8,10^, which highlights the fundamental need of understanding the biological and molecular mechanisms underlying statin sensitivity.

By blocking the mevalonate pathway, statins will reduce the level of intracellular cholesterol and trigger a compensatory response, which attempts to replenish it, by increasing intracellular cholesterol synthesis and uptake from the circulation, via mechanisms including upregulation of *HMGCR* and low density lipoprotein receptor (*LDLR*) expression^1,11^. Although this mechanism is proven to work in non-cancer cells, especially hepatocytes^1,2^, statin-induced effects on the regulation of LDL-cholesterol trafficking and eventually on lipid metabolism in BC cells are still poorly understood. Preclinical data support the hypothesis that the anticancer activity of statins would likely be more effective against primary breast tumors and BC cell lines bearing a dysregulation in the “cholesterol biosynthesis” process^8,10,12,13^. In this regard, we recently showed that the extent of upregulation of cholesterol biosynthesis genes, including *HMGCR*, by atorvastatin was weaker in treatment-sensitive BC cells compared to the insensitive counterparts^5,10^. Moreover, we identified a cholesterol biosynthesis gene signature, whose differential expression at baseline could potentially predict statin-sensitivity in BC primary tumors and cell lines^10^.

Indeed, higher basal transcript levels of this signature, which included *HMGCR*, predicted low sensitivity to statin therapy and correlated with poor prognosis after primary breast cancer diagnosis^10^, irrespective of estrogen receptor alpha (ER) status. However, a complete understanding of the molecular mechanisms underlying statin response in BC is still necessary to enable the identification and development of highly specific response biomarkers for clinical utility.

Some evidence indicates that basal levels of LDL-cholesterol and lipid metabolism are not uniform across BC cells. A lipid-accumulating phenotype was observed in the ER- basal-like MDA-MB-231 cells, which appeared to have a more accentuated storage of neutral lipids like triglycerides and cholesterol derivatives or esters as cytoplasmic lipid droplets (LDs), when compared to the luminal-like ER^+^ MCF7 cells^14,15^. Recent data also suggests a role for LDs as a ready–to-use dynamic lipid source for relevant biological processes, including energy production, synthesis of new cell membranes^16^, modulation of nuclear functions, cell signaling, cell survival and lipid metabolism in general^14,17–19^. The lipid accumulating phenotype was also characterized by increased LDL uptake in addition to lower *HMGCR* activity^15^ and higher *LDLR* mRNA expression^20^ at baseline. The effect of statin treatment on this lipid accumulating phenotype in breast cancer cells is however poorly described.

In light of the differences observed in basal lipid metabolism levels, we sought to further investigate how atorvastatin affects intracellular lipid regulation in BC cells and whether this effect, if any, was associated with the anti-proliferative response to the treatment. Our results provide additional molecular insight into the associations between lipid metabolism and the response of BC cells to statin therapy, moving a step further towards unravelling the molecular mechanisms underlying the role of statins in preventing breast cancer progression.

## Results

### Atorvastatin-induced cell growth inhibition is heterogeneous across BC cell lines

We have previously reported that the anti-proliferative effects of statins on breast cancer cell lines is largely dependent on the expression of the ER, with very potent effects observed in ER negative cell lines^10^. To verify our previous results, a similar panel of BC cell lines were exposed to increasing doses of atorvastatin for 72 hrs and thereafter were classified into two groups, namely; statin-sensitive and -insensitive cells, according to the magnitude of the growth inhibitory effect. We elected to use the lipophilic statin, atorvastatin, given its favorable pharmacokinetics properties^21^ together with the minimum side effects, observed in our previously conducted pre-operative clinical trial, when using the maximum dose of 80 mg/daily prescribed to optimize the likelihood of statin delivery to BC tumors^6^. As expected, T47D and MCF-7 cells (both ER+/PR+/HER2−) appeared less sensitive to statin treatment as they required atorvastatin concentrations higher than 5 μM to significantly inhibit cell growth (inhibition rate more than 50%) **(**Supplementary Fig. S1**)**. On the other hand, MDA-MB-231 cells (ER−/PR−/HER−) were classified as extremely sensitive because of the potent inhibitory effects on cell proliferation already at doses corresponding to 1 μM **(**Supplementary Fig. S1**)**. Likewise, BT474 (ER+/PR+/HER2+) and SKBR3 (ER−/PR−/HER2+) cell lines were classified as insensitive and moderately sensitive respectively **(**Supplementary Fig. S1). These results are remarkably consistent with our previous report (supplementary fig. S1B in^10^) and largely align with data from other studies evaluating the anti-proliferative response of BC cell lines to statin treatment^5,8^.

### Atorvastatin triggers progressive accumulation of intracellular LDs in statin-insensitive BC cells

**Figure 1:**
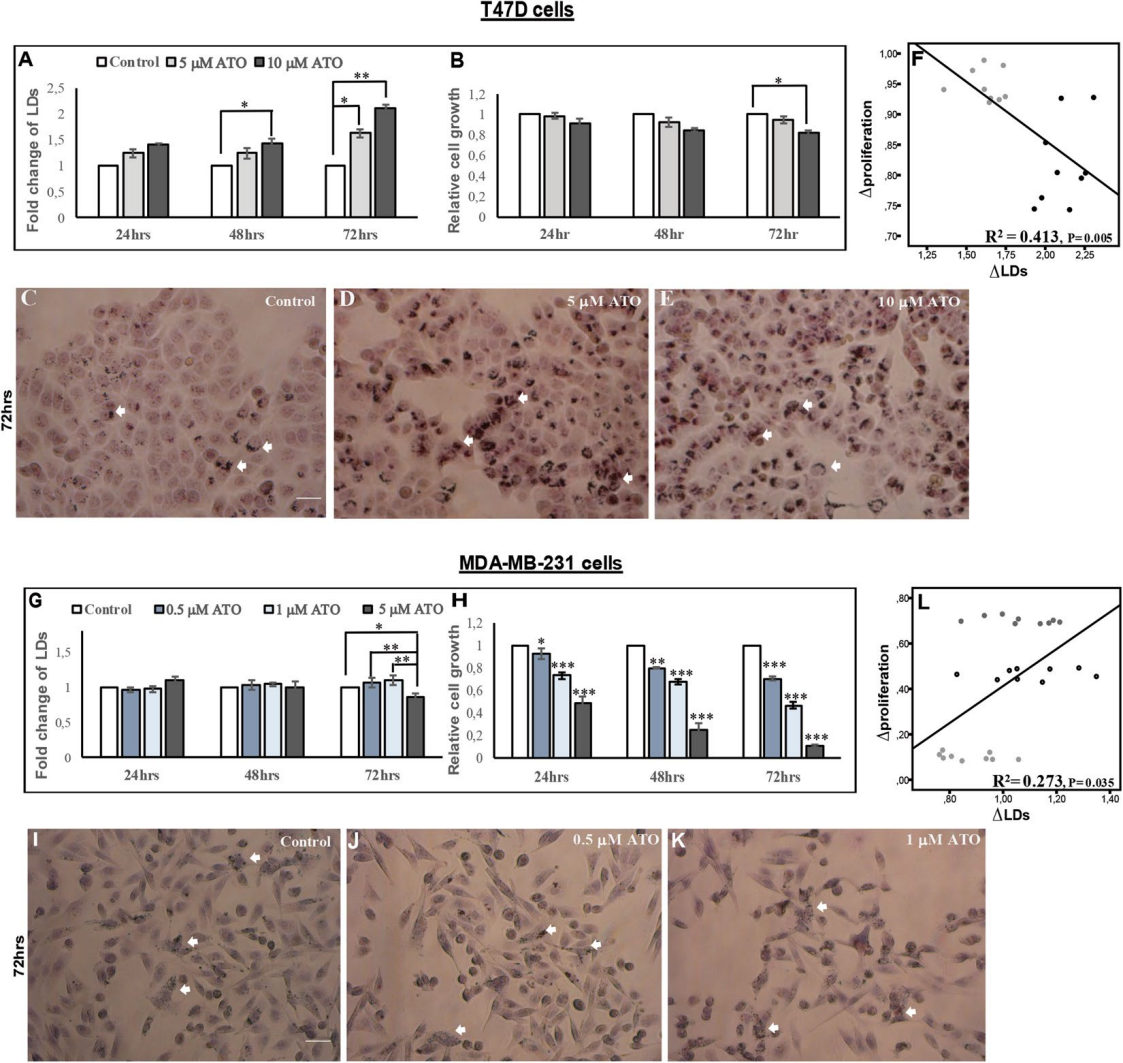
Atorvastatin induced differential effects on LD accumulation in insensitive T47D cells and sensitive MDA-MB-231 cells. BC cells were incubated over time with vehicle-(DMSO) or increasing doses of atorvastatin (ATO) up to 72hrs and LD content was evaluated. (**A**) and (**G**) depict a summary of LD content in T47D and MDA-MD-231 cells, respectively. The anti-proliferative effects of atorvastatin were assessed in parallel: (**B**) and (**H**) for T47D and MDA-MB-231, respectively. Data were collected from three independent experiments and *error bars* indicate SD. * represents P < 0.05; ** represents P < 0.01 and *** indicates P < 0.001. Associations between fold changes (Δ) in cell proliferation and LD abundance are shown for insensitive T47D cells **(F)** and for sensitive MDA-MB-231 cells **(L)** following 72hrs of exposure to atorvastatin. T47D cells were treated with 5 μM (grey circles) and 10 μM (black circles) of atorvastatin **(F)** and MDA-MB-231 cells were treated with 0.5 μM (dark grey circles), 1 μM (black circles) and 5 μM (light grey circles) atorvastatin **(L**), respectively. Representative images of LD staining by Oil Red O were acquired following 72hrs atorvastatin exposure in T47D cells **(C–E)** and MDA-MB-231 cells **(I–K)**. *White arrows* indicate examples of intracellular Oil Red O-stained LDs in BC cells. *Scale bars* correspond to 50 μm.

As statins inhibit the HMGCR enzyme, and in turn block cholesterol biosynthesis, we aimed to evaluate if atorvastatin altered intracellular lipid levels and whether these effects may differ according to the anti-proliferative response to the treatment. Our results showed that there was a differential capability of storing neutral lipids between the sensitive and insensitive BC cells already at baseline **(**Supplementary Fig. S2A). The sensitive MDA-MB-231 cells appeared significantly more prone to accumulate LDs compared to the insensitive T47D and MCF7 cells by 1.5 folds and 3.8-folds, respectively **(**Supplementary Fig. S2A; adjusted p < 0.01 for all comparisons). Following treatment with increasing doses of atorvastatin ranging up to 10 μM, the relative number of LDs increased over time in the insensitive T47D cells as compared to untreated controls **(**Fig. 1A**)**. A dose-dependent rise in LD levels, which was markedly pronounced following 72hrs of exposure to atorvastatin (fold changes in LDs; 1.62 (p < 0.05) and 2.11 (p < 0.01) for 5 μM and 10 μM doses, respectively) was observed **(**Fig. 1A,C–E**)** and this rise in LD abundance appeared to inversely mirror the size of the inhibitory effect of atorvastatin on cell growth (p < 0.05, Fig. 1A,B and F). A similar trend was observed in MCF7 cells **(**Supplementary Fig. S2B). In contrast, no significant increase in LD biogenesis was observed in MDA-MB-231 cells following incubation with increasing doses of atorvastatin up to 1 μM over 72hrs **(**Fig. 1G,I–K**)**, despite the corresponding dramatic impairment of cell proliferation **(**Fig. 1H**)**. Following 72hrs exposure to 5 μM atorvastatin, a significant decrease in LD levels paralleled the very potent anti-proliferative effects **(**Fig. 1G,H and L**)**. A weak positive correlation between atorvastatin-induced relative change in LD biosynthesis and inhibition of cell growth was noted after 72hrs incubation time (p < 0.05; Fig. 1L).

### Atorvastatin induces differential expression of genes involved in lipid metabolism between treatment-sensitive and insensitive BC cells

**Figure 2:**
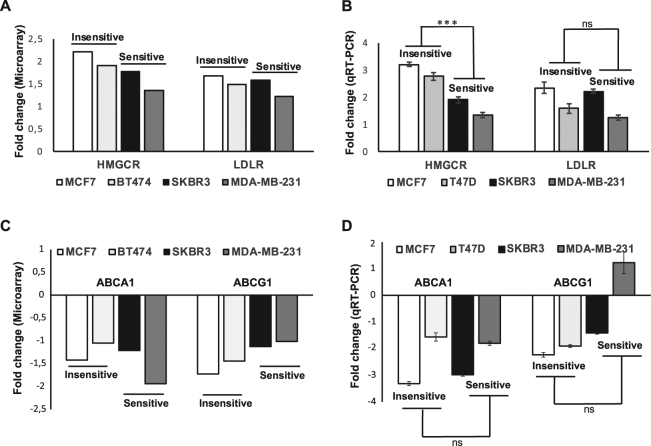
Relative expression of cholesterol metabolism regulators following atorvastatin treatment in BC cells. Fold changes in the expression of *HMGCR*, *LDLR*, *ABCA1* and *ABCG1* mRNA were assessed in BC cells following 48hrs exposure to atorvastatin by microarrays (**A** and **C**) and validated by qRT-PCR (**B** and **D**). Results were obtained from two independent experiments conducted in triplicate with *errors bars* indicating SD. ** represents P < 0.01 and *ns* indicates not significant difference respectively.

In our previous study, we reported that atorvastatin triggered significant upregulation of *HMGCR* and several other genes involved in the cholesterol biosynthesis pathway, a sub-pathway within lipid metabolism^5,10^. Importantly, we noted that the fold difference in HMGCR expression was significantly greater in the less-sensitive cell lines^10^. We therefore investigated whether the atorvastatin-triggered differential effects observed on LD accumulation could also be associated with concomitant differences in the relative expression of key genes associated with lipid metabolism between the two groups. Initially, we compared the transcript levels of *HMGCR* (regulator of cholesterol biosynthesis) and *LDLR* (cholesterol uptake), and *ABCA1* and *ABCG1* (involved in cholesterol efflux and maintenance of cellular cholesterol homeostasis and confirmed to be expressed in breast cancer) between treatment-insensitive (MCF7, BT474, T47D) and sensitive (SKBR3 and MDA-MB-231) cell lines following 48hrs exposure to atorvastatin. Atorvastatin treatment upregulated the relative transcript levels of *HMGCR* and *LDLR* in all cell lines by microarray analyses **(**Fig. 2A**)** and these results were confirmed by qRT-PCR **(**Fig. 2B**)**. As expected, the magnitude of *HMGCR* upregulation was significantly higher in the group of insensitive BC cells compared to the sensitive counterparts (p < 0.001; Fig. 2B) confirming our hypothesis that very strong induction of *HMGCR* expression after statin exposure is a marker of treatment resistance^10^. Conversely, although atorvastatin-induced *LDLR* expression in all cell lines (Fig. 2A–B and Supplementary Fig. S3A for relative differences in *LDLR* expression between cell lines and treatments), no significant fold difference was evident between the two groups (p = 0.207; Fig. 2B). However, *LDLR* expression was relatively significantly higher in MDA-MB-231 cells compared with all other cell lines both at baseline and following statin treatment (Supplementary Fig. S3A).

Furthermore, statin treatment resulted in a significant downregulation of at least one of the reverse cholesterol transporters; *ABCA1 and ABCG1*, in BC cell lines. Specifically, the mRNA levels of *ABCA1* were significantly downregulated in all BC cells in response to atorvastatin **(**Fig. 2C,D and Supplementary Fig. S3B for relative *ABCA1* expression**)**, but similar to *LDLR*, the extent of downregulation could not consistently discriminate between treatment-sensitive and insensitive BC cells **(**p = 0.735; Fig. 2D**)**. In addition, the very sensitive MDA-MB-231 cells displayed the highest expression of *ABCA1* both at baseline and following atorvastatin treatment (Supplementary Fig. S3B). *ABCG1* was also downregulated is all cell lines except MDA-MB-231 (Fig. 2C and D) but the average fold difference between the sensitive and insensitive groups was statistically insignificant (p = 0.372; Fig. 2D).

Since the levels of *HMGCR* expression and LD accumulation were significantly differentially altered by atorvastatin in a somewhat consistent trend between the sensitive and insensitive BC cells, we performed a SAM analysis to further explore whether these observations could be extended to include any other gene(s) involved in the global lipid metabolism pathway (GO:0006629), given the null associations observed for the specific investigations for *LDLR*, *ABCA1* and *ABCG1* expression and response to treatment.

**Figure 3:**
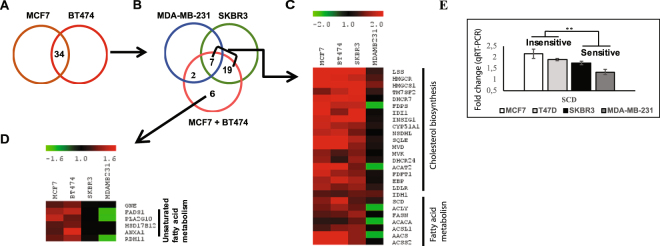
Atorvastatin induced differential expression of genes involved in lipid metabolism between insensitive and sensitive BC cells. SAM analyses comparing atorvastatin treated (48hrs) and DMSO/controls in insensitive MCF7 and BT474 cells resulted in 34 genes commonly upregulated by atorvastatin **(A)**. 7 out of these 34 genes were consistently upregulated in all four BC cells, while 19 genes were upregulated in the insensitive (MCF7 and BT474) BC cells and mildly sensitive SKBR3 cells only **(B)**. “Cholesterol biosynthesis” and “Fatty acid metabolism” processes were biological processes enriched among the 34 genes upregulated by atorvastatin, although the fold increases were smaller in the sensitive BC cells **(C)**. Red and green in the heatmap represents up-and downregulation respectively. “Unsaturated fatty acid metabolism” process was exclusively upregulated in insensitive BC cells **(D)**. Fold changes in transcript levels (measured by qRT-PCR) of SCD, key regulator of unsaturated fatty acid biosynthesis, appeared consistently lower in the sensitive (SKBR3-MDA-MB-231) cells **(E)**. Results were obtained from two independent experiments conducted in triplicate with *errors bars* indicating SD. ** represents P < 0.01.

After SAM analyses and setting an arbitrary fold change (FC) cut off of > 1.2, we identified 34 genes that were commonly upregulated in the insensitive BC cells (MCF7 and BT474) **(**Fig. 3A**)**, 26 of which were also found to be upregulated in the moderately sensitive (SKBR3) cells, and only 9/34 were upregulated in the extremely sensitive MDA-MB-231, as indicated in the Venn diagram **(**Fig. 3B**)**. While 7 out of the 34 genes overlapped between all four cell lines, 6 genes were exclusively altered in the insensitive cell line **(**Fig. 3B**)**. Gene ontology (GO) analysis revealed that the “cholesterol biosynthesis”, “super-pathway of cholesterol biosynthesis” and “activation of gene expression by SREBP” were the top ranking biological processes and sub-pathways in this selected set of 34 genes **(**Table 1**)**. Indeed, 16 out of 34 genes, including *HMGCR* itself, were involved in the “cholesterol biosynthesis” process showing significantly higher transcript expression in the insensitive (MCF7 and BT474) cells compared to the moderately sensitive SKBR3 and the extremely sensitive MDA-MB-231 cells **(**Fig. 3C**)**. Further, atorvastatin treatment appeared to mildly, yet statistically significantly increase the expression of 7 genes involved in fatty acid metabolic pathway and transport to a greater extent in insensitive cells compared to the sensitive BC cell group **(**Fig. 3C, Table 1**)**. Interestingly, among the 6 genes found to be exclusively associated with insensitive cells, 4 genes (HSD17B12, FADS1, PLA2G10, ANXA1) were involved in the biological process of “metabolism of unsaturated fatty acids” **(**Fig. 3D**)**, which has a fundamental role in providing basic building blocks, such as unsaturated fatty acids, of more structurally complex lipids in mammalian cells^22^. In addition, the transcript level of stearoyl COA desaturase (*SCD*), which catalyzes the rate limiting step in the synthesis of unsaturated fatty acids, and more generally lipid synthesis^22^, was significantly lower in the sensitive (SKBR3 and MDA-MB-231) BC cells compared to the insensitive (MCF7 and BT474) cells (Fig. 3C). Given that SCD is a key regulator of lipid synthesis, qRT-PCR was performed to validate the microarray results. Similarly, qRT-PCR analyses revealed that statin treatment upregulated SCD in all cell lines with significantly higher fold-increases in the insensitive cells (P < 0.01; Fig. 3E and Supplementary. Fig. S3C) compared to the sensitive cells. Taken together, these results suggest that the dysregulation of not only cholesterol biosynthesis but possibly fatty acid metabolism, specifically the synthesis of unsaturated fatty acids, are likely associated with response to atorvastatin therapy.

**Table 1:** Gene ontology analysis identified biological processes and pathways altered by atorvastatin treatment in a set of 34 differentially expressed genes involved in lipid metabolism.

Biological Processes	P value	FDR	In data	Gene list
Cholesterol biosynthetic process (GO:0006695)	**4,51E-23**	**9,15E-21**	**16**	INSDHL1, SQLE, MVD, ACAT2, LSS, MVK, DHCR24, LSS, HMGCS1, DHCR7, HMGCS1 FDFT1,CYP51A1,IDI1,FDPS,TM7SF2.
Fatty acid metabolic process (GO:0006631)	**2.46E-13**	**1.40E-11**	**12**	**HSD17B12,ANXA1, FADS1, PLA2G10,SCD**, FASN, ACCS2,ACACA,ACLY, AACS, ACSL1, INSIG.
Fatty acid biosynthetic process (GO:0006633)	**1.00E-12**	**5.45E-11**	**9**	**HSD17B12, ANXA1, FADS1, SCD**, FASN, ACCS2, ACACA,ACLY,INSIG1.
Unsaturated fatty acid metabolic process (GO:0033559)	**9.93E-7**	**2.65E-5**	**5**	**ANXA1, FADS1,PLA2G10,SCD**,ACSL1.
Long-chain fatty acid transport (GO:0015909)	**2.11E-6**	**5.48E-5**	**4**	**ANXA1, PLA2G10**,ACSL1,ACACA.
**Pathways**	**P value**	**FDR**	**In data**	**Gene list**
Cholesterol biosynthetic	**1,21E-26**	**2,93E-24**	**14**	INSDHL1, SQLE, MVD, ACAT2, FDFT1, FDPS, CYP51A1, IDIL, DHCR24, DHCR7, LSS, HMGCR, HMGCS1,TM7SF2.
Superpathway of cholesterol biosynthesis	**2,90E-24**	**3,53E-22**	**15**	TM7SF2, LSS, HMGCR, HMGCS1, IDI1, FDPS, DHCR24, IDIL, FDFT1, CYP51ANSDHL1, SQLE, MVD, ACAT2, DHCR7.
Regulation of Cholesterol biosynthesis by SREBP	**5,52E-18**	**1,49E-16**	**15**	TM7SF2, LSS, HMGCR, HMGCS1, IDI1, NSDHL1, SQLE, MVD, ACAT2, FDFT1, FDPS, DHCR24,DHCR7, CYP51A1, IDIL.
Fatty acid metabolism	**6,63E-10**	**5,04E-09**	**7**	**HSD17B12, FADS1, SCD**, ACSL1, FASN, ACACA, ACAT2.
Fatty acid biosynthesis	**3.79E-11**	**2.72E-10**	**6**	**SCD**, ACSL1, FASN, ACSS2,ACACA, ACLY.
Biosynthesis of unsaturated fatty acids	**2.41E-5**	**1.375E-4**	**3**	**HSD17B12, FADS1,SCD**.

## Discussion

Despite the advances in adjuvant chemo- and endocrine therapies, breast cancer is still one of the leading causes of death worldwide^23^. Lipophilic statins represent a promising candidate for therapeutic interventions in breast cancer due to the robust association found between statin use and reduced risk of cancer-related mortality^24^ and prevention of breast cancer recurrences^3,4,25^. So far, an increasing body of literature has demonstrated the heterogeneous capability of statins to affect key biological processes supporting cancer progression, likely via inhibiting cell cycle/proliferation and increasing apoptosis^6,8,10,26,27^.

It is still unclear whether the anticancer activity of lipophilic statins is likely dependent on the direct effects of statins on BC cells or if it is linked to their systemic lipid lowering function, which ultimately confers indirect effects prohibiting breast cancer growth^8,10,28,29^. In the present study, we have investigated the direct effects of atorvastatin on regulation of BC lipid metabolism and corresponding changes in intracellular lipid levels in a panel of BC cell lines representing different levels of responsiveness to the treatment. We set the sensitivity threshold at an atorvastatin concentration ≤5 μM, which is a reported dose range likely to be clinically achieved in the circulation after oral statin administration^30^. Since the impact of statins on BC lipid metabolism has been poorly investigated, our study represents a valid starting point to shed light on this important subject.

We have shown that in the presence of an exogenous supply of cholesterol and fatty acids, inhibition of HMGCR activity with atorvastatin triggers a progressive dose-dependent accumulation of neutral lipids specifically in the BC cells displaying little or no anti-proliferative response to the treatment (resistance). The amount of stored LDs is possibly greater than is required for anabolic or catabolic processes. On the other hand, in the extremely sensitive MDA-MB-231 cells, the capability to produce LDs was not altered over time by lower doses of atorvastatin (ranging up to 1 μM) despite the corresponding strong inhibition of cell growth. Nevertheless, the extremely potent anti-proliferative effects caused by higher concentrations of atorvastatin (5 μM) in MDA-MD-231 cells were paralleled by a significant decrease in the relative LD content. Neutral lipids can be accumulated in response to stress stimuli^31–33^ and serum lipoproteins and/or fatty acids are fundamental sources for LD biogenesis, when cells are maintained in optimal culture conditions^16^. However, the exact functional role of LDs in breast cancer progression, especially in response to statin treatment, is largely unknown. A possible interpretation of this finding is that while the insensitive cells adopt a lipid-accumulating phenotype to counteract the stress conferred by statins on the cholesterol biosynthesis and lipid metabolic pathways, the sensitive cells undergo a dramatic impairment in cell growth, which culminates in a metabolic state where the rate of neutral lipid consumption is likely higher than the rate of storage, as the cells try to survive the potent statin-induced stress. Although we did not specifically quantify the effect of treatment on the total cholesterol content, the fact that cholesterol esters is a major component of LDs suggests that a parallel increase in total cholesterol content in the insensitive cells is a likely consequence of the increase in LD content. The source of the extra cholesterol/lipids generating the LDs is plausibly from uptake from extracellular sources since in the presence of statins, the catalytic activity of HMGCR necessary for *de novo* cholesterol biosynthesis is inactivated despite significant upregulation of the negative-feedback loop. However, other factors may be associated with this complex phenotype hence further studies including lipidomic analyses are warranted to shed more light into this complex phenotype. Nonetheless, if validated, this LD accumulating phenotype can serve as an early biomarker of resistance to statin treatment and therefore a valuable addition to clinical trials investigating statins use as breast cancer therapy.

Statins are competitive inhibitors of cholesterol biosynthesis. Hence, we hypothesized that the atorvastatin-induced differential effects on neutral lipid accumulation observed between sensitive and insensitive BC cells could likely reflect differences in the statin-induced feedback mechanism, which regulates the expression of *LDLR* and *HMGCR* in accordance to the levels of intracellular cholesterol (lipids)^1,13^. Gene expression analyses by both microarrays and qRT-PCR confirmed that atorvastatin triggered an upregulation of *HMGCR* and *LDLR* in all BC cells, suggesting an induction of the compensatory feedback response, a finding which is consistent with previous investigations^8,10^. Interestingly, we found through qRT-PCR analyses that the magnitude of *LDLR* upregulation was lower in the extremely sensitive MDA-MB-231 cells relative to the insensitive BC cells. However considering that the basal *LDLR* expression in MDA-MB-231 cells was already relatively higher compared to the other cell lines, this may suggest that greater gene upregulation was not necessary since the relative *LDLR* levels in MDA-MB-231 post atorvastatin treatment was still higher than in any other cell line. Accordingly, dysregulation of LDLR expression has recently been suggested to make prostate cancer cells more likely to respond to statin treatment and to mediate statin-induced inhibitory effects on cell growth^34^. One potential interpretation of our findings can be related to the intrinsic lipid accumulating phenotype of the basal-like/ER- cells, including MDA-MB-231 cells^10^, which may indicate that maximal, or near- maximal LDLR-mediated uptake of lipids from the culture medium, and consequently LD accumulation, already exists at baseline. Indeed, the very high basal *LDLR* expression in MDA-MB-231 was paralleled by higher basal LD abundance relative to the other cell lines. In addition, statin-induced downregulation in *ABCA1 and ABCG1*; mediators of cellular cholesterol and lipid export necessary for maintaining cellular cholesterol homeostasis, was found to be similar between the groups of sensitive and insensitive BC cells. In fact, despite being downregulated by the treatment in all cell lines, *ABCA1* expression was still the highest in MDA-MB-231 displaying little or no change in LD content. However, acetyl-CoA acetyltransferase 2 (*ACAT2*), which is involved in the excretion of lipoproteins containing cholesterol esters from human intestine, fetal liver and leukocytes^35^ was found to be selectively upregulated in the less sensitive cells (MCF7, BT474, SKBR3, Fig. 3C) suggesting the likelihood that attempts to prevent toxic levels of lipid accumulation by increasing efflux channels following statin exposure may still be active in the less sensitive cells. Our results lend more support to the hypothesis that differences in the regulation of lipid uptake through mechanisms involving *LDLR* expression might better explain the inability of atorvastatin to induce an additional accumulation of neutral lipids above baseline in the very sensitive cells, which may have impacted treatment responsiveness. However, when the moderately sensitive SKBR3 cells were included in the analysis, we did not observe a consistent pattern for the atorvastatin-induced dysregulation of *LDLR* transcript expression within the entire group of sensitive BC cells, although their belonging to two distinct clinical subtypes, HER2+ and triple negative-subtype respectively^36,37^, may be a plausible explanation for such variable response to treatment.

As LDs are composed not only of cholesterol derivatives but also of triglycerides, we extended our investigation to explore how atorvastatin affected the global expression of genes involved in lipid metabolism. Notably, atorvastatin induced a more marked upregulation of *HMGCR* in insensitive BC cells compared to the sensitive counterparts consistent with our previous findings that dysregulation of *de novo* cholesterol biosynthesis and *HMGCR* expression is a better predictor of responsiveness to statin therapy in cell lines and patient tumors^10^.

For the first time, we have identified *SCD* as an additional gene significantly upregulated to higher levels in insensitive BC cells compared to the sensitive counterparts after atorvastatin exposure. SCD is the rate limiting enzyme for the synthesis of monounsaturated fatty acids (MUFAs) from endogenous or exogenous sources of saturated fatty acids (SFAs)^38,39^. Some data indicates that MUFAs are likely the preferential substrates for the formation of the major lipid species in mammalian cells (e.g., phospholipids, cholesteryl esters, triglycerides and their derivatives)^22,40^. Accordingly, “fatty acids metabolism” appeared to be a mildly but still significantly differentially expressed biological process following atorvastatin exposure among the two groups of BC cells. Notably, upregulation in “unsaturated fatty acid biosynthesis and metabolic pathway” was also exclusively associated with statin insensitivity in our analyses. Moreover, genetic and pharmacological ablation of SCD has been shown to significantly decrease proliferation and negatively impact survival of breast cancer cells^22,41,42^. These data likely support the hypothesis of a potential involvement of SCD in the tight regulation of fatty acid, or global lipid biosynthesis, and cancer cell growth^22^, although its functional link to cancer progression still needs to be thoroughly investigated. Further, no data are available regarding the impact of lipophilic statins on SCD expression, activity and more generally on fatty acid metabolism in BC cells. Nevertheless, SCD levels are controlled by regulatory-element-binding-proteins (SREBPs), which are the same family of transcription factors responsible for statin-induced regulation of *HMGCR* expression^38^. Although further analyses on atorvastatin-induced SCD protein expression are reasonably required, our results indicate a potential involvement of cholesterol biosynthesis and fatty acid metabolism in the response mechanisms associated with statin treatment. Interestingly, co-targeting SREBP-mediated regulation of the compensatory feedback response triggered by statin therapy has previously been proposed as a promising strategy to potentiate the antitumor activity of lipophilic statins in breast cancer^43^.

In summary, we speculate that atorvastatin-induced LD biogenesis may represent an initial attempt to protect BC cells from the potential toxicity of an intracellular excess of either free fatty acids, like SFAs^22,44^, or cholesterol. Accumulation of LDs may in turn act as ready-to-use catabolic fuel^32^ for overcoming drug-induced stress conditions. However, when the stress stimulus becomes severe and the formation of LDs likely exceeds the maximal storing capacity of BC cells, this phenomenon is accompanied by a parallel dramatic effect on BC cell proliferation.

Future functional studies are now required to confirm this hypothesis and unravel the likely contributions of SREBP-mediated regulation of cholesterol-feedback mechanism and fatty acid metabolism on LD formation. This will clarify the potential role of neutral lipids in the antitumor activity of lipophilic statins in breast cancer. Importantly, in addition to *HMGCR*, this study highlights that rapid accumulation of intracellular LDs and strong induction of SCD mRNA expression are potentially novel biomarkers of statin resistance in BC cells. Pre-clinical and clinical studies to identify in greater depth, key biomarkers within either cholesterol biosynthesis or unsaturated fatty acid metabolism, which may have a functional role in mediating breast cancer responsiveness to statin therapy, are highly recommended.

## Material and Methods

### Cell lines, culture conditions and cell treatments

Human breast cancer (BC) cell lines [T47D, MCF7, BT474, SKBR3 and MDA-MB-231] were purchased from ATCC (Rockville, MD) and asynchronously grown at 37 °C and 5% CO_2_ humidified atmosphere. T47D cells were maintained in RPMI 1640 medium and the remaining cell-lines were grown in culture media as described previously [10], supplemented with 10% (v/v) fetal bovine serum (FBS-HyClone^TM^) and with an antibiotic cocktail made of 100 U/ml penicillin and 100 mg/ml streptomycin. Atorvastatin calcium salt trihydrate (Sigma Aldrich) was dissolved in DMSO prior to cell treatments. To determine sensitivity, BC cell lines were exposed to increasing concentrations of atorvastatin (1–50 μM) for 72 hrs and the effects on cell growth were assessed by using xCelligence Real Time Cell Analysis (RTCA) instrument (ACEA Bioscience, Inc).

### Oil Red O staining

When testing progressive effects of atorvastatin on neutral lipid storage in the form of LDs, BC cells were exposed to increasing concentrations of atorvastatin for up to 72hrs. T47D (5–10 μM) and MDA-MB-231 (0,5–5 μM), served as representative models for statin insensitive and sensitive BC cells respectively. Cell fixation with 3% paraformaldehyde (PFA) in PBS was followed by a pre-incubation step in 60% isopropanol prior to the final staining with filtered Oil Red O-working solution (60% of Oil Red O main stock solution (Sigma Aldrich) and 40% of deionized water). A series of washing steps in 60% isopropanol, 10% isopropanol and PBS were conducted to rinse away excess dye. Empty wells were stained in parallel and used for background correction. Neutral lipid-bound Oil Red O dye was extracted from the stained cells using 100% isopropanol and a semi-quantitative measurement of the LD abundance was performed by measuring the absorbance at 518 nm wavelength using the automatic FLUOstar OPTIMA multi-detection microplate reader (BMG Labtech). Data were adjusted based on atorvastatin-induced inhibitory effects on cell growth rate, which was measured in parallel by Sulforhodamine B (SRB- Sigma) proliferation assay. All experimental conditions were run in triplicate. Results were expressed as relative change in the amount of LDs in treated over control cells in three independent experiments ± standard deviation (SD) of the mean. Also parallel to the semi-quantitative assessment of LD abundance, nuclei were counterstained with hematoxylin following Oil-Red-O staining and representative images were taken using Zeiss Axiovert 40 CFL inverted microscope supplemented by Canon Powershot G9 camera.

### Sulforhodamine B (SRB) - cell proliferation assay

For cell growth analysis performed in parallel with LD measurements, BC cells were exposed to same treatments as used for Oil Red O staining. Fixed cells were then stained with 0.4% (w/v) SRB in 1% acetic acid for 20 min and the unbound dye was removed by sequential washes with 1% acetic acid. The protein-bound dye was then dissolved in 10 mM Tris base and the absorbance measured via the FLUOstar OPTIMA microplate reader at 570 nm wavelength. Semi-quantitative measurements of statin-induced cell growth inhibition was performed over untreated cells and conducted in three separate experiments. Data were presented as relative cell growth ± SD.

### RNA isolation and microarray data analysis

For gene expression analysis, MCF7 cells were treated with 5 μM of atorvastatin and MDA-MB-231 with 1 μM of atorvastatin for 48hrs in complete culture conditions. BT474 and SKBR3 were also introduced in the analyses, as additional insensitive and sensitive BC models respectively, and were also exposed to 5 μM of atorvastatin. Total RNA was extracted using RNA mini kit (Qiagen) according to the manufacturer’s instructions. The RNA quality and integrity were determined via an Agilent 2100 Bioanalyser (Agilent) and RNA quantification was accomplished using NanoDrop ND-1000 (NanoDrop Products). RNA amplification, labelling and hybridization were performed onto the Human HT-12 v4.0 Expression BeadChips according to the Illumina-recommendations (Illumina, Inc.). The gene expression data can be assessed from the Gene Expression Omnibus (GEO) under the accession number GSE63427. The microarray data processing and normalization, exclusion criteria for underperforming probes together with results describing the effects of statin treatment on global gene expression and cholesterol metabolism in particular have been reported in our previous studies^5,10^. For the current investigation, we have restricted the transcriptional analyses to genes involved in “lipid metabolic process; GO:0006629”. 1162 of the 1265 genes, included in the gene ontology term GO:0006629, were present on the microarray and were included in the analyses. Independent Significant Analysis of Microarray (SAM) analyses comparing untreated controls and statin-treated samples for each cell line was conducted using TMev 4.9 software in order to identify lipid metabolism genes differentially altered by atorvastatin treatment in each cell line. Data were further filtered to exclude genes showing fold changes <1.2 and the cut-off for statistical significance was set at a false discovery rate (FDR) <0.05. The genes remaining after applying these filtration thresholds underwent Gene Ontology (GO) classification to investigate if any sub-molecular functions, biological processes or sub-pathways within lipid metabolism were significantly enriched for among the genes altered by atorvastatin. GO terms were investigated in human specific databases by using the online portal “ToppGene Suite”, https://toppgene.cchmc.org/.

### Quantitative real time PCR (qRT-PCR)

Cells were exposed to the same experimental procedures, in terms of atorvastatin treatment and total RNA was isolated, prior to relative gene expression quantification by qRT-PCR. cDNA was synthesized from 1 μg of total RNA using High-Capacity cDNA Reverse Transcription kit (Thermo Fisher Scientific). To confirm the data from whole genome transcriptional profiling of canonical regulators of lipid and cholesterol metabolism, qRT-PCR was performed using TaqMan QuantiTect Probe PCR Kit (Quiagen) together with predesigned primer/probe sets (Applied Biosystem) to amplify *HMGCR* (Hs00168352_m1), *LDLR* (Hs01092525_m1), *ABCA1* (Hs01059118_m1), *SCD* (Hs01682761_m1). *B-actin* or *ACTB* (Hs99999903_m1) was used as reference gene. Expression of all transcripts, as threshold cycle (Ct) value, was measured in triplicate and normalized to the housekeeping gene, *ACTB*; relative levels of mRNA expression in atorvastatin-treated compared to untreated cells were subsequently determined by 2-ΔΔCt method^45^ in two independent experiments. Samples containing no transcripts were used as negative controls.

### Statistical analysis

To test for atorvastatin-induced differences in cell growth and accumulation of LDs between atorvastatin-sensitive and -insensitive BC cells, we performed one-way ANOVA with post-hoc comparisons using Bonferroni’s method to adjust for multiple testing. Differential atorvastatin-induced effects on the mRNA levels of regulators of cholesterol biosynthesis and lipid metabolism between statin-sensitive and insensitive groups of BC cells were assessed by the student’s t-test. The association between the relative atorvastatin-induced effects on proliferation and neutral lipid storage capacity of BC cells was evaluated by using Spearman’s correlation analysis. Statistical analyses were performed using using IBM SPSS Statistics 24 software and the level of significance was set at p < 0.05.

## Electronic supplementary material

Supplementary figures S1-S3
