# Supplementary material for: Insensitivity to atorvastatin is associated with increased accumulation of intracellular lipid droplets and fatty acid metabolism in breast cancer cells
Source: Sci Rep. 2018 Apr 3;8:5462. doi: 10.1038/s41598-018-23726-3 (PMC5882899; doi:10.1038/s41598-018-23726-3)

Insensitivity to atorvastatin is associated with increased accumulation of intracellular lipid droplets and fatty acid metabolism in breast cancer cells

Barbara Lettiero, Maria Inasu, Siker Kimbung and Signe Borgquist

Supplementary Information

Supplementary Fig. S1: Differential atorvastatin-induced anti-proliferative effects on BC cells. BC cells were treated with increasing doses of atorvastatin (1-50μM) for 72hrs. The growth rate of each BC cell line upon statin exposure relative to controls is shown**.** Data were collected from three independent experiments and *error bars* represent SD of the mean.

Supplementary Fig. S2: (A) Basal LD content of BC cells is associated with sensitivity to statin treatment. LD abundance in untreated MCF7, T47D and MDA-MB-231 cells was quantified by measuring the absorbance (518 nm) after Oil Red O staining. Data plotted are mean ± SD of three independent measurements. ** $P<0.01$. **(B) Atorvastatin-induced change in LD biosynthesis is inversely correlated with its anti-proliferative effects in MCF7 cells.** MCF7 cells were exposed to increasing atorvastatin doses, specifically 5 μM (grey circles) and 10 μM (black circles) for 72hrs and effects on proliferation and LD biosynthesis were measured in parallel. Association between relative growth inhibition (Δ) and relative abundance (Δ) of LDs was evaluated by Spearman´s correlation analysis. Experiments were conducted in triplicate and data were obtained from three independent experiments.

Supplementary Fig. S3: Relative differences in mRNA expression levels of **(A)** *LDLR*, **(B)** *ABCA1* and **(C)** *SCD* between BC cell lines and treatment conditions. The expression of each target gene is normalized to the expression of the house keeping gene *(ACTB*) and the data are presented as ΔCT. All target genes were expressed at lower levels relative to the *ACTB* for all conditions hence lower ΔCT values indicates higher relative expression and vice versa. Data plotted are the mean ΔCT values and standard deviations from at least two independent qRT-PCR experiments.

Supplementary Fig. S1

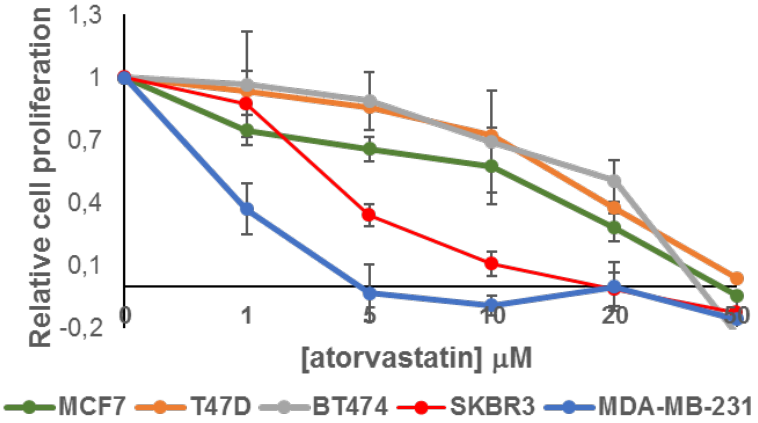

Supplementary Fig. S2

A

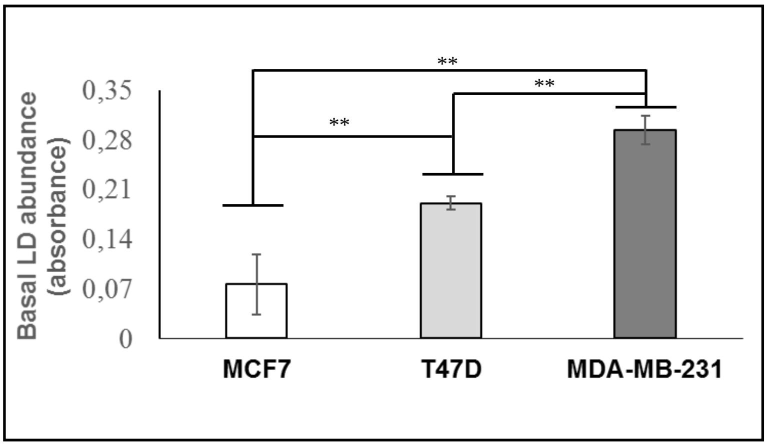

B

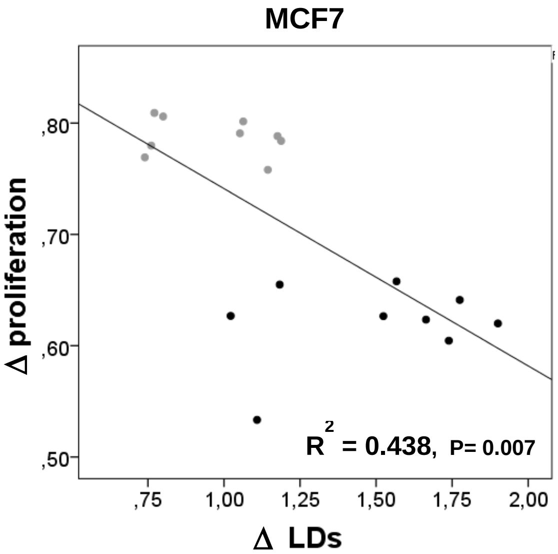

Supplementary Fig. S3

A

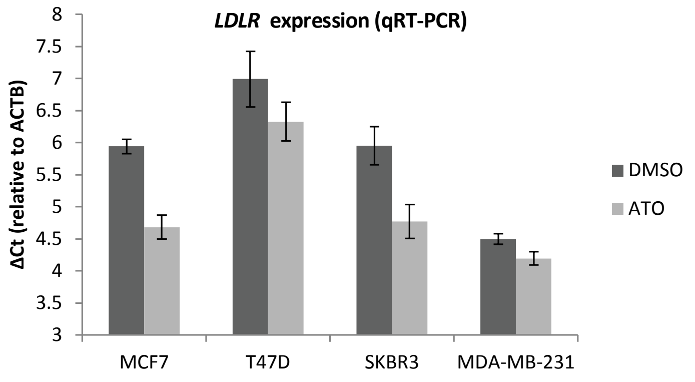

B

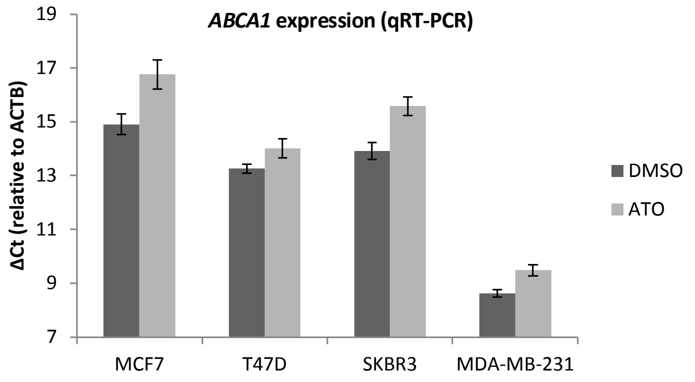

C

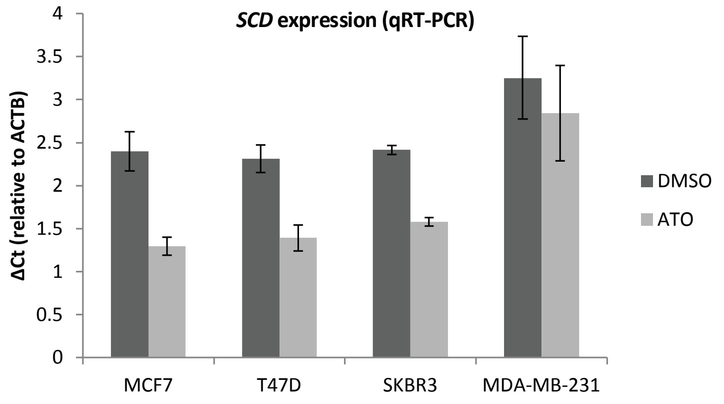

Supplement: Supplementary file 1 — Supplementary figures S1-S3 [file 41598_2018_23726_MOESM1_ESM.pdf]
